# Self‐Assembled Flexible and Integratable 3D Microtubular Asymmetric Supercapacitors

**DOI:** 10.1002/advs.201901051

**Published:** 2019-08-26

**Authors:** Fei Li, Jinhui Wang, Lixiang Liu, Jiang Qu, Yang Li, Vineeth Kumar Bandari, Daniil Karnaushenko, Christian Becker, Maryam Faghih, Tong Kang, Stefan Baunack, Minshen Zhu, Feng Zhu, Oliver G. Schmidt

**Affiliations:** ^1^ Material Systems for Nanoelectronics Chemnitz University of Technology 09107 Chemnitz Germany; ^2^ Center for Materials Architectures and Integration of Nanomembranes (MAIN) Chemnitz University of Technology 09126 Chemnitz Germany; ^3^ Institute for Integrative Nanosciences Leibniz IFW Dresden 01069 Dresden Germany

**Keywords:** 3D microtubular architecture, footprints, integrated devices, microsupercapacitors, rolled‐up nanotechnology

## Abstract

The rapid development of microelectronics has equally rapidly increased the demand for miniaturized energy storage devices. On‐chip microsupercapacitors (MSCs), as promising power candidates, possess great potential to complement or replace electrolytic capacitors and microbatteries in various applications. However, the areal capacities and energy densities of the planar MSCs are commonly limited by the low voltage window, the thin layer of the electrode materials and complex fabrication processes. Here, a new‐type three‐dimensional (3D) tubular asymmetric MSC with small footprint area, high potential window, ultrahigh areal energy density, and long‐term cycling stability is fabricated with shapeable materials and photolithographic technologies, which are compatible with modern microelectronic fabrication procedures widely used in industry. Benefiting from the novel architecture, the 3D asymmetric MSC displays an ultrahigh areal capacitance of 88.6 mF cm^−2^ and areal energy density of 28.69 mW h cm^−2^, superior to most reported interdigitated MSCs. Furthermore, the 3D tubular MSCs demonstrate remarkable cycling stability and the capacitance retention is up to 91.8% over 12 000 cycles. It is believed that the efficient fabrication methodology can be used to construct various integratable microscale tubular energy storage devices with small footprint area and high performance for miniaturized electronics.

## Graphical abstract

**A type of three‐dimensional (3D) tubular asymmetric microsupercapacitors** with small footprint area, high potential window, ultrahigh areal energy density, long‐term cycling stability, and good flexibility, is fabricated with shapeable materials and photolithographic technologies. The design provides an efficient counterpart of conventional macroscopic cylinder‐shape energy storage devices at the microscale, showing promising potential for microelectronic applications.

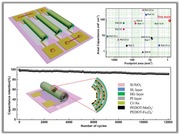

Along with the rapid development of modern industry, recent technological trends toward portable and smart electronics, such as remote and mobile environmental sensors, microelectromechanical systems, microrobots, and implantable medical devices, have continuously increased the demands for miniaturized energy storage devices (MESDs).^1^, ^2^ Among them, planar microsupercapacitors (MSCs) have received much attention owning to their superior power density, excellent cycling stability, fast rate capabilities, wide working temperature range, desirable safety properties, and minimal maintenance costs.^3^

To date, MSCs can be classified into three categories, namely, sandwich type, interdigital type, and fibrous type, depending on the configuration of electrodes used.^4^, ^5^ The interdigital structures and their flexibility in electrode design provide attractive advantages by avoiding narrowing interspaces between electronically isolated electrode fingers, preventing short circuits, and minimizing internal impedance.^6^, ^7^ However, the areal capacities and energy densities of the in‐plane MSCs are commonly limited by the low voltage window and the small thickness of the electrode materials. For instance, current planar MSC systems still suffer from low areal capacity: ≤11.6 mF cm^−2^ for carbons,^2^, ^8^ ≤78 mF cm^−2^ for conducting polymers,^9^ and ≤58 mF cm^−2^ for metal oxides.^10^, ^11^ Thus, it is still a big challenge to efficiently and cost‐effectively fabricate asymmetric MSCs with high areal capacities and energy densities.

An optimal strategy to tackle the above challenges is to build asymmetric MSCs using two different electrode materials to expand the operating potential window and thus increase the energy density. Therefore, recent efforts have been employed to explore novel pseudocapacitive cathode and anode materials to achieve large specific capacitance and good rate performance. For the cathode, transition metal oxides such as MnO_2_,^10^ Co_3_O_4_,^12^ RuO_2_,^13^ and V_2_O_5_ 14 dominate the landscape of active materials for electrochemical energy storage. MnO_2_ electrodes have significant benefits such as low cost, abundant resources, stable crystal structure, environmental compatibility, and high theoretical specific capacitance (1370 F g^−1^). On the other hand, iron oxides/hydroxides (α‐Fe_2_O_3_, β‐Fe_2_O_3_, γ‐Fe_2_O_3_, Fe_3_O_4_, α‐FeOOH, β‐FeOOH, γ‐FeOOH, and δ‐FeOOH) have received tremendous interest as promising anode materials among their many competitors for asymmetric supercapacitors (ASCs) because of the multiple valences of iron, rich redox chemistry in the negative potential window, low cost, and abundant resources.^15^ However, both MnO_2_ and iron oxides/hydroxides exhibit poor electrical conductivity, insufficient ionic diffusion rate, volume expansion, and severe aggregation during the redox reactions, which often lead to inadequate utilization and pulverization of the active materials. Tremendous efforts have been devoted to address this issue, including: (1) use of nanostructures, such as nanoparticles, nanotubes, nanowires, and nanoflakes to provide short transport paths for ions, highly active contact areas, and effective buffers during charge–discharge processes.^16^ (2) Combination of nanostructures with conductive materials including carbon‐based materials and conducting polymers.^17^ However, carbon coatings, which are usually realized by thermal decomposition of carbon precursors, can cause environmental problems because of the formation of volatile compounds, such as CO and CO_2_. Conductive polymer coating is therefore a good alternative for the enhancement of conductivity and stability of MnO_2_ and iron oxides/hydroxides.

Although asymmetric MSCs can be fabricated by inkjet printing, laser writing, and silicon‐based molding technologies, MSCs still suffer from cumbersome manufacturing procedures and low areal energy densities, inhibiting their scalable production and further applications.^4^, ^18^ One critical factor for asymmetric MSCs is that the device footprint area should be kept as small as possible in order to cater potential applications in microelectronic circuits and microsystems. Obviously, integrating more active material into cleverly designed three‐dimensional (3D) electrode architectures will effectively increase the areal performance within a limited footprint area. Therefore, to develop smart and reliable fabrication methodologies for 3D integrated asymmetric MSCs with high areal energy densities has become an urgent and decisive task in the field of MSCs. Among various efforts, rolled‐up tubular micro‐/nanostructures which are fabricated by a simple origami self‐assembly approach provide an attractive and unique route for the fabrication of energy storage devices. For instance, large area planar capacitor membranes can self‐assemble into “Swiss‐roll” architectures,^19^ thereby improving the areal performance and space utilization in electronic circuitry. Meanwhile, the cylinder shell provides appropriate fixation and efficient mechanical protection based on these developments, 3D cylinder microstructures represent a powerful and promising strategy for constructing high performance integrated 3D asymmetric MSCs.

Here, we demonstrate a fully integrated asymmetric MSC with ultrahigh areal energy density and excellent cycling performance fabricated by self‐assembly approach utilizing shapeable materials technologies.^20^ Our fabrication route which is compatible to microelectronic manufacturing processes provides a promising methodology to develop fully integratable energy storage devices. A novel electrode material system is developed, in which MnO_2_ and Fe_3_O_4_ nested in poly(3,4‐ethylenedioxythiophene) (PEDOT) layers are working as cathode and anode, respectively. The nanostructures in these electrodes not only supply sufficient electrochemically active sites on the surfaces of MnO_2_ and Fe_3_O_4_, but also increase the effective liquid–solid interfacial areas, causing fast paths for the insertion and extraction of electrolyte ions, and consequently facilitating the Faraday reaction. Owing to the robust fixation by the 3D microtubular structures and the much decreased footprint area, the asymmetric MSC exhibits a wide electrochemical potential window of 1.5 V, an improved specific areal capacitance of 88.6 mF cm^−2^ at 0.66 mA cm^−2^, an ultrahigh areal energy density of 28.69 µWh cm^−2^ and power density of 39.47 mW cm^−2^, as well as a very long‐term cycling stability (retention of 91.5% after 12 000 cycles), superior to all previous works based on MnO*_x_* and FeO*_x_*/Fe(OH)*_x_* materials.

The whole device fabrication procedures toward the asymmetric MSCs are schematically illustrated in **Figure** **1**a. At first, the shapeable material layer stack^21^, ^22^ is patterned starting with 400 nm thick lanthanum acrylate based sacrificial layer (SL) patterned on a Si/SiO_2_ substrate followed by a 900 nm thick photocrosslinked hydrogel‐based swelling layer. Afterward, a 1700 nm thick patterned polyimide (PI) layer as reinforcement layer was fabricated to cover the hydrogel layer, as shown in Figures 1a and **2**a. The details of the sacrificial layer, hydrogel layer, polyimide layer, and their patterning are described in the Experimental Section. Subsequently, standard lithography and electron beam evaporation were used to pattern the interdigital current collectors on the polyimide layer, as shown in Figures 1a and 2b. 10 nm Cr and 50 nm Au thin films were successively deposited to form interdigital finger electrodes. The finger width and gap are 200 and 50 µm, respectively. In order to comfortably perform electrodeposition of the anode and cathode, printed circuit boards (PCBs) were used to connect the contact pads of the MSCs interdigital electrodes with the standard three electrodes of an electrochemical work station. Then, electrodeposition of PEDOT/MnO_2_ and PEDOT/Fe_3_O_4_ was accomplished, respectively. The self‐assembly into the Swiss‐roll architecture was performed in potassium diethylenetriaminepentaacetic acid solution with pH = 10, which is able to selectively etch the sacrificial layer. The hydrogel layer swells when absorbing the etching solution, while the polyimide layer without the swelling property maintains its original shape. As a result, the bilayers are released from the substrate when the sacrificial layer is etched away, and the strain between the hydrogel and polyimide layers causes the rolling to proceed. It is worth pointing out that the hydrogel layer was designed into a trapezoidal shape (**Figure** **2**a) so that the area close to the longer edge of the hydrogel layer has a larger strain. A strain gradient along the “Y” direction was formed and the rolling direction was well defined. By placing the samples into the etching solution, the sacrificial layer was selectively dissolved, leading to controllable rolling of the planar devices into multiwound microtubes containing polymeric layer stacks, metallic finger electrodes, and electrode material films. During the etching process, the cross‐linked hydrogel layer swells as carboxyl groups uptake a large amount of etching solution. However, the PI layer is inert to the external stimuli due to its stable chemical properties. This strain mismatch between the bilayer generates the self‐assembly force and detaches the whole structure from the substrate, thus demonstrating controllable and directional reshaping of planar devices. After taking out from the etching solution, the etching solution remains in the hydrogel layer, leading to a continuous rolling force.

**Figure 1:**
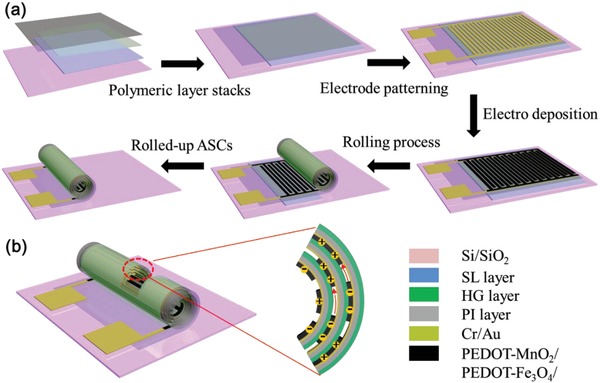
a) Schematic illustration of the design and fabrication of rolled‐up asymmetric MSC. b) Conceptual scheme of a rolled‐up tubular MSC. Negative ion transport is indicated by red arrow when charging process takes place inside the tubular device.

**Figure 2:**
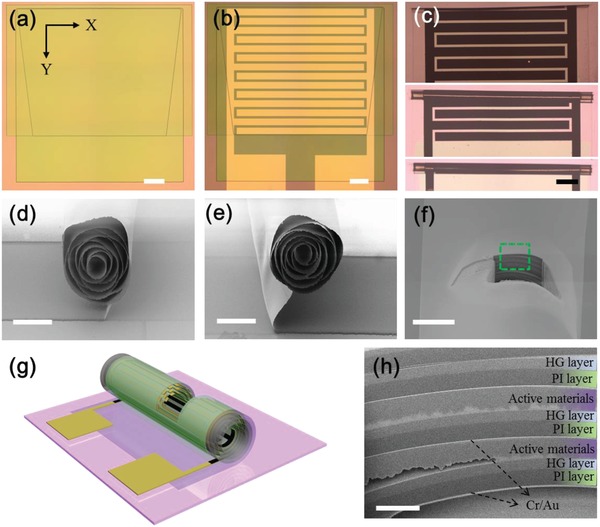
a–c) Optical microscope images of the three layers, Cr/Au current collectors, electrodeposited materials, and the rolling process (scale bar (a–c): 500 µm). d,e) SEM images of tube openings, which were taken from two ends of the tube (scale bar (d,e): 100 µm). f–h) Schematic illustration and SEM images of the tube cross‐section prepared by FIB‐cutting (scale bar (f): 50 µm, scale bar (h): 5 µm).

The rolling processes of MSCs on SiO_2_/Si and PI substrates at different stages were recorded in Figure 2c. The PEDOT–MnO_2_ and PEDOT–Fe_3_O_4_ and metal electrodes were rolled up from the top edge into multiple windings and will finally form a compact tubular MSC after about 12 h. As shown in Figure 2c and Figure S3 (Supporting Information), the tube diameters range from 180 to 220 µm. The yield of tubular devices achieves more than 90%. In Figure 2d,e, the scanning electron microscopy (SEM) images show the two open ends of a tubular structure with multiple windings with a diameter of about 200 µm. The outer part of the tube wall was cut by a focused ion beam (FIB) to reveal the internal structure (see Figure 2f,h). This cut shows several outside windings of the “Swiss‐roll” architecture, which consists of the thick polymeric bilayer (composed of the hydrogel layer and polyimide layer; total thickness ≈2.7 µm), bright current collector, and active materials film (≈2–2.5 µm). As shown in Figure 2h, the thickness of each layer remains unchanged in the three windings, indicating good homogeneity of the polymeric bilayer and a constant deposition rate over large areas of the thin film materials. The tubular devices maintained their shape when they were taken out of the etching solution, which greatly facilitates the operation of MSCs.

The structure of the cathode (PEDOT–MnO_2_) and anode (PEDOT–Fe_3_O_4_) materials on the Cr/Au film and the design of an MSC are schematically illustrated in **Figure** **3**a. The electrodes are formed by the electrochemical growth of active materials, MnO_2_ nanoforests and Fe_3_O_4_ nanosheets, on the as‐deposited porous PEDOT nanonetworks. These novel hierarchical structures of active materials demonstrate superior surface areas than bulk materials and compact membranes and provide fast paths for the insertion and extraction of electrolyte ions.

**Figure 3:**
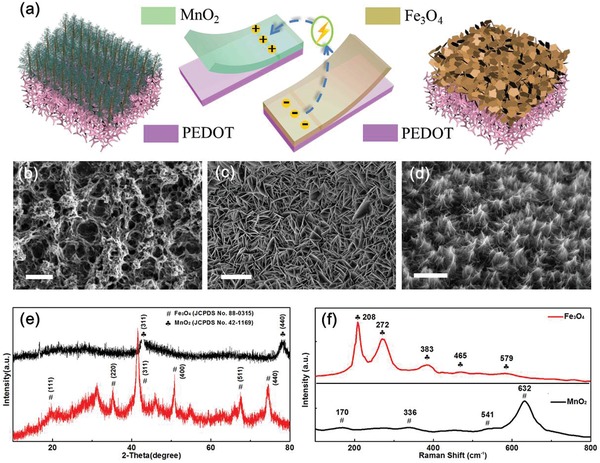
a) Schematic illustration of the active materials. b–d) SEM images of PEDOT, PEDOT–Fe_3_O_4_, and PEDOT–MnO_2_ (scale bar (b): 500 nm, scale bar (c): 2 µm, scale bar (d): 500 nm). e) XRD patterns of MnO_2_ and Fe_3_O_4_. f) Raman spectra of MnO_2_ and Fe_3_O_4_ thin films.

The SEM image in Figure 3b shows a representative morphology of the PEDOT thin films. The porous structure of the PEDOT facilitates the infiltration of the electrolyte ions. The SEM images in Figure 3c and Figure S4a (Supporting Information) reveal that the Fe_3_O_4_ nanosheets grow vertically and are cross‐linked on the PEDOT layer. The dense Fe_3_O_4_ film consists of regular nanosheets, typically 0.5–2 µm in length and 30–50 nm in thickness, nested with the PEDOT networks. X‐ray diffraction (XRD) is employed to investigate the crystalline structures and phase purity of the as‐prepared Fe_3_O_4_. As shown in Figure 3e, the diffraction peaks in the pattern can be indexed to the reported data for Fe_3_O_4_ (JCPDS card no. 88–0315). The diffraction peaks at 2θ = 21.32°, 35.17°, 41.49°, 50.58°, 67.42°, and 74.34° are assigned to the plane (111), (220), (311), (400), (511), and (440), respectively. The sharp and narrow diffraction peaks indicate that the Fe_3_O_4_ thin films possess high crystallinity. Transmission electron microscopy (TEM) images collected from the Fe_3_O_4_ nanostructure also verify its nanosheet morphology, as shown in Figure S5a,b of the Supporting Information.

Figure 3d and Figure S4b (Supporting Information) show the SEM images of MnO_2_ nanoforests, which embed in the PEDOT networks. Figure 3e presents the composition and crystallite phase purity of the MnO_2_ nanoforests. The diffraction peaks are observed at 43.31° and 78.02°, which are assigned to the plane (311) and (440) of MnO_2_, respectively (JCPDS card no. 42–1169). The detailed structural information of MnO_2_ is further provided by TEM (Figure S5c,d, Supporting Information), which indicates that the MnO_2_ nanoforests consist of small stacked ultrathin MnO_2_ nanosheets. During the electrochemical deposition on current collectors, the lateral outward growth of the electrode materials is much smaller than the gap between the two finger electrodes (50 µm). Therefore, the short circuit problem can be avoided.

The crystal structures of the samples were then studied by Raman spectroscopy, as shown in Figure 3f. The Raman spectrum collected from the as‐prepared MnO_2_ sample has four bands located at 170, 336, 541, and 632 cm^−1^, which are characteristic for the MnO_2_ phase. The strong bands around 600–650 cm^−1^ are due to symmetrical Mn–O vibrations and indicate the well‐developed cubic structure. The Raman peaks located at 208, 272, 383, 465, and 579 cm^−1^ can be indexed to the spectroscopic modes of the Fe_3_O_4_ phase.

Before assembling into the integrated 3D tubular MSCs, the electrochemical behavior of the cathode and the anode materials were first investigated in a three‐electrode configuration in 1 m LiCl solution, as shown in Figures S1 and S2 of the Supporting Information. The PEDOT–MnO_2_ and PEDOT–Fe_3_O_4_ were directly used as the working electrodes. A platinum plate was used as the counter electrode and a saturated calomel electrode was taken as the reference electrode. As there is a wide potential difference between PEDOT/MnO_2_ and PEDOT/Fe_3_O_4_, these electrode materials can be assembled into a hybrid asymmetric MSC. PVA/LiCl gel was used to seal the tube structures to attain the long‐term operation of the tubular devices.

Figure S6 of the Supporting Information shows cyclic voltammetry (CV) curves of the tubular device in different potential windows. A working potential window of 1.6 V was considered to be the maximum and beyond which some irreversible reactions may occur. Thus, a safe and optimum working potential window from 0 to 1.5 V was employed for this asymmetric MSC. **Figure** **4**a shows the CV curves of the tubular MSC at various scan rates between 20 and 100 mV s^−1^ with a potential window of 0–1.5 V. A couple of redox peaks are observed from 0.4–0.6 V to 0.65–0.8 V, indicating a pesudoreaction from the electrodes during the charge–discharge process. The fast and reversible successive surface redox reactions of MnO_2_ are caused by intercalation/deintercalation processes of the protons according to reaction (1). The pseudocapacitance reaction of Fe_3_O_4_ may result from the redox reactions between Fe^2+^ and Fe^3+^ accompanied by intercalation of chloride ions balancing the extra charge with the iron oxides (2)

(1) $${\mathrm{MnO}}_{2}+H^{+}+{\mathrm{Li}}^{+}+2e^{-}↔\mathrm{MnOOHLi}$$

(2) $$2{\mathrm{Fe}}^{\mathrm{II}}O+{\mathrm{Cl}}^{-}↔\left({\mathrm{Fe}}^{\mathrm{III}}O\right)+{\mathrm{Cl}}^{-}\left({\mathrm{Fe}}^{\mathrm{II}}O\right)+e^{-}$$

**Figure 4:**
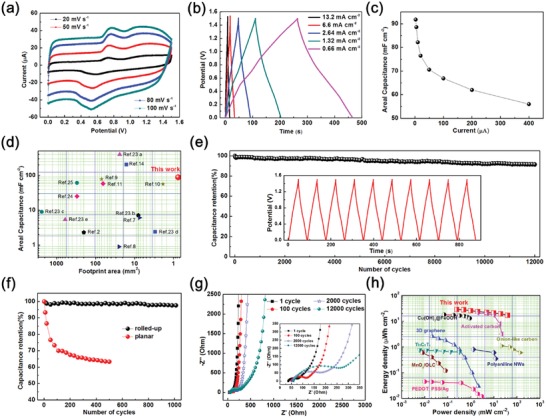
a) CV curves with different scan rates. b) GCD curves of MSCs at various current densities. c) Specific areal capacitance under different current densities. d) Comparison of the areal capacitance and the footprint area of tubular MSCs with reported interdigital MSCs. e) Cycling performance of the device. Inset shows the last ten GCD curves of the 12 000 cycles. f) Comparison of the cycling performance between the 3D tubular device and the corresponding planar device. g) Electrochemical impedance spectra (EIS) of the 3D tubular MSCs after different cycles. h) Ragone plots comparing the 3D tubular MSCs with carbon‐based MSCs, metal oxide‐based MSCs, and conducting‐polymer‐based MSCs.

In addition, the CV curves display a nearly rectangular shape in a potential window from 0–0.2 V to 1.2–1.5 V, reflecting the main electrochemical characteristics of PEDOT. As calculated from the galvanostatic charge–discharge (GCD) curves (Figure 2b), outstanding areal capacitances up to 88.6 mF cm^−2^ were achieved at 0.66 mA cm^−2^, with a retention of about 75.5% (66.9 mF cm^−2^) when the current density was increased to 13.2 mA cm^−2^. More galvanostatic charge–discharge tests of the MSC devices are conducted, and the rate capability is shown in Figure 4c. The areal capacitance retained more than 55 mF cm^−2^ at a high current density of 52.48 mA cm^−2^, showing the desirable rate performance. The high rate capability is attributed to the high conductivity of the electrodes and small charge transfer resistances (as shown in Figure 4g). In addition, with the more and more intense demands on miniaturization of electronic devices, many MSCs which possess a footprint area larger than 10 mm^2^ cannot be easily integrated into a microelectronic system and connected with other microdevices on the same chip. Therefore, it is challenging but desirable to develop high performance integrated MSCs with a minimal footprint area. Figure 4d and Table S1 (Supporting Information) compare the areal capacitance of tubular MSCs with other recent reports. Our tubular MSCs possess much smaller footprint areas (less than 0.8 mm^2^) than most of the reported works,^2^, ^7^, ^8^, ^9^, ^10^, ^11^, ^14^, ^23^, ^24^, ^25^ and deliver a comparatively higher areal capacitance of 88.6 mF cm^−2^.

The cycling stability of the device is further investigated by galvanostatic charge/discharge cycling between 0 and 1.5 V at 2.64 mA cm^−2^ (as shown in Figure 4e). The specific capacitance retained 91.8% after 12 000 cycles, indicating its good cycle stability. The optical microscope images of the tubes after long term cycling are shown in Figure S7 of the Supporting Information. The tubes keep their tubular structures and diameters well after cycling. The charge–discharge curves of the last 10 cycles of the 12 000 cycles are shown as the inset in Figure 4e. It can be seen that the curves reveal a highly linear and symmetric shape with small resistance drops, further confirming the excellent supercapacitive behavior and superior electrochemical reversibility. In addition, the cycling stability within 1000 cycles of the MSC before and after rolling was compared in Figure 4f. The retention of the planar device drops down sharply due to the poor adhesion of the electrode materials on the substrate during cycling. It is probably caused by the excessive stress of the thick planar electrodes fabricated by long‐term electrodeposition. However, the self‐wound polymeric nanomembrane templates mitigate the stress, and tightly fix and protect the thick active materials. The rational design concept of the Swiss‐roll architecture for interdigital electrodes can be extended to other applications, which can pave the way for safety and implantable electronic devices.

Figure 4g shows Nyquist plots of the asymmetric MSC after different cycles measured in a frequency range from 100 kHz to 0.1 Hz. The corresponding equivalent circuit consists of a series and parallel combination of resistances, *R*_s_ (contributions of the ionic resistance of the electrolyte, intrinsic resistance, and contact resistance between the active material and the current collector), *R*_ct_ (charge transfer resistance), CPE (constant phase element), and W (Warburg impedance). The charge transfer resistance (*R*_ct_) change from 41 to 260 Ω after 12 000 cycles calculated by the diameter of the semicircles in high‐frequency regions, which is related to reaction kinetics. Only a slight increase of the internal resistances (*R*_s_) from 35.9 to 59.7 Ω is observed after 12 000 cycles, manifesting a good conductivity of the electrolyte and the very low internal resistance of the electrode.

Unlike for traditional supercapacitors, where electrochemical performances are normalized to mass, it is the areal performance such as the areal capacitance, areal energy and power densities, which is important for benchmarking microenergy storage units against the limited footprint area available for on‐chip microdevices and microsystems. Therefore, the areal energy densities of the as‐fabricated tubular MSCs and other very recently reported MSC energy storage devices are summarized in a Ragone plot (Figure 4h) and Table S1 (Supporting Information) for comparison. The maximum energy density obtained for our asymmetric tubular MSCs is 28.69 µW h cm^−2^ at a power density of 0.25 mW cm^−2^. Even at a high power density of 39.47 mW cm^−2^, the MSC can deliver a high energy density of 17.13 µW h cm^−2^, which exceeds those of previously reported MSCs,^8^, ^11^, ^24^, ^25^, ^26^, ^27^ including both carbon‐based electrical double‐layer MSCs and pseudo‐MSCs, such as 2.6 µW h cm^−2^ for a 3D laser‐scribed graphene,^24^ 18.07 µW h cm^−2^ for an Cu(OH)_2_@FeOOH/Cu MSC,^11^ and 0.6 µW h cm^−2^ for an MnO_2_/OLC MSC.^26^

In order to satisfy the specific energy requirements for higher voltage and capacitance in practical applications, multiple 3D asymmetric MSCs based on PEDOT/MnO_2_ and PEDOT/Fe_3_O_4_ active electrode materials are interconnected in series and parallel, as shown in **Figure** **5**a,b. The configuration of MSCs in parallel shows an increased capacitance value for two and three cells compared to that of a single cell. As shown in Figure 5c, when the asymmetric MSCs are connected in parallel (two or three devices), the charge/discharge time for the parallel connected devices (at a voltage window of 1.5 V) is increased by two or three times over that of a single device, respectively. In the case of two or three MSCs interconnected in series, the CV voltage window is increased to 3 or 4.5 V, twice or three times higher than that of a single device (Figure 5d). Furthermore, two ASCs are interconnected in series to light up a commercial timer (rated voltage ≈ 1.5 V) and a green light‐emitting‐diode (LED) (rated voltage ≈ 1.9 V) (Figure 5e,f). The LED was running for about 20 s after the MSCs were charged for 5 s. The higher capacitance and larger operating voltage window achieved in the MSCs by simple parallel and serial interconnections facilitate their practical application as energy storage devices.

**Figure 5:**
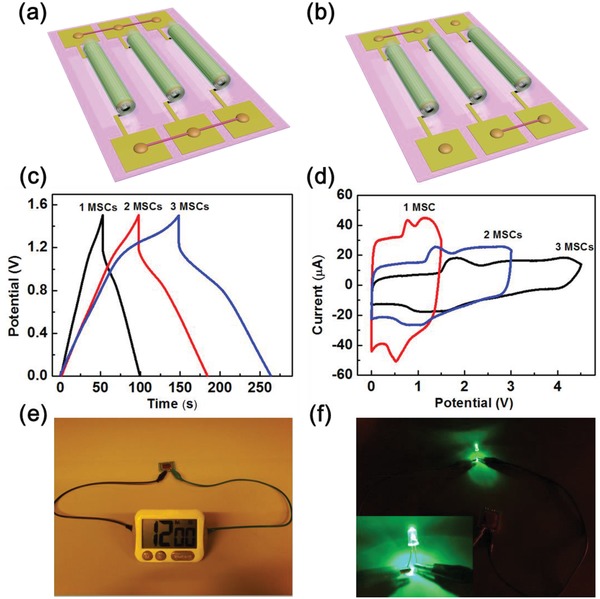
a,b) Schematic illustration of tubular devices interconnected in series and parallel. c) GCD curves of single‐tube MSCs interconnected in parallel. d) CV curves of single‐tube MSCs interconnected in series. e,f) Photographs of tubular MSCs in series powering a timer and an LED.

To meet the future demands of the portable and flexible electronics, such as roll‐up displays, electronic paper, wearable devices, and microrobots, it is required to develop flexible, light‐weight MSC systems with large energy and power density. However, 3D MSCs with advantageous flexibility have been rarely reported, as shown in Table S2 of the Supporting Information.^14^, ^23^, ^28^ In comparison, our tubular MSCs show excellent mechanical performance without compromise in their electrochemical storage properties. As shown in **Figure** **6**a,b, the freestanding tubular MSCs were transferred onto a flexible PI substrate. The performance of the flexible samples was tested under different deformations. During bending and twisting from 0° to 90° and from 0° to 60°, respectively, the CVs of the tubular MSC devices retain their shapes and remain almost unchanged even at a high scan rate of 200 mV s^−1^, indicating that the 3D tubular MSCs possess remarkable stability and excellent flexibility under bending and twisting (Figure 6c,d). Therefore, the tubular devices can be easily integrated into various flexible and stretchable chips or electronic textiles, paving the way for potential applications of MSCs as flexible energy storage devices in portable, stretchable, and/or wearable electronic devices.

**Figure 6:**
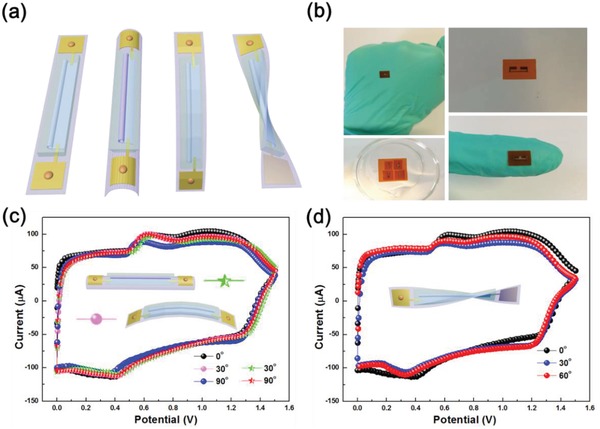
a) Illustrations of different deformations. b) Digital photographs of the MSCs on flexible substrate. c,d) CV measured at a scan rate of 200 mV s^−1^ for the MSCs subjected to (c) bending and (d) twisting.

We have demonstrated novel on‐chip 3D tubular asymmetric MSCs based on PEDOT–MnO_2_ anode and PEDOT–Fe_3_O_4_ cathode materials employing Swiss‐roll architecture and shapeable materials technologies. The rolled‐up self‐assembly process can effectively reduce the footprint area of the original planar devices, greatly improving the areal specific capacitance (88.6 mF cm^−2^ at 0.66 mA cm^−2^) and areal energy density (28.69 mW h cm^−2^ at 0.25 mW cm^−2^). Moreover, the cycling stability of 3D tubular MSCs showed remarkable capacitance retention up to 91.8% over 12 000 cycles. In addition, the tubular MSCs show excellent mechanical performance without sacrificing their electrochemical storage properties. Our work demonstrates a new powerful strategy to create highly integrated MSCs with high performance and small footprint area. The 3D tubular MSC provides an efficient counterpart of conventional macroscopic cylinder‐shape energy storage devices at the microscale, and shows promising potential for microelectronic applications.

## Experimental Section

*Fabrication of Rolled‐Up MSCs: Polymeric Layer Stack*: The solution of the polymeric layer stack including sacrificial layer and strained bilayers was synthesized as previous reported.^21^ For preparation of the polymeric layers, the sacrificial layer solution was first spin‐coated onto the O_2_ plasma‐cleaned Si/SiO_2_ substrates at 6000 rpm for 50 s. The samples were then baked at 40 °C for 10 min for solvent removal and further exposed (365 nm, 15 W cm^−2^) for 4 min through a glass/Cr photomask using an SUSS MJB4 mask aligner (Karl Suss KG‐Gmbh&Co, Munchen‐Garching, Germany). The patterned samples were developed in deionized water for 1.5 min and finally baked at 220 °C for 30 min to obtain stable sacrificial layer.

Second, the solution for swelling hydrogel layer was spin‐coated at 6000 rpm for 90 s. After baking at 40 °C for 10 min, samples were exposed (365 nm, 15 W cm^−2^) for 0.5 min through the glass/Cr photomask using SUSS MJB4 mask aligner. The second layer was developed in 2‐(2‐Methoxyethoxy) ethanol (Sigma‐Aldrich) for 2 min and rinsed in propylene glycol methyl ether acetate (microresist technology GmbH) to remove unexposed polymer. Finally, the samples were baked at 220 °C for 30 min, resulting in a layer with thickness of about 900 nm.

Polyimide with the thickness of about 1600 nm was prepared as a nonswelling layer on hydrogel layer. In detail, polyimide solution was spin‐coated at 4000 rpm for 90 s and baked at 50 °C for 10 min. After exposed (365 nm, 15 W cm^−2^) for 1.5 min through the glass/Cr photomask, the samples were then developed in a mixture of 1 part (v/v) of DMSO and 9 parts (v/v) of propylene carbonate (Sigma‐Aldrich, Germany) until the unexposed layer with color disappeared. Finally, the samples were rinsed in propylene glycol methyl ether acetate and baked at 220 °C for 30 min.

*Interdigital Electrodes*: Standard photolithography was used to achieve patterned photoresist (AZ 5214E) on the polymeric layers coated substrate. And then, 10 nm Cr and 50 nm Au were successively deposited using e‐beam evaporator (Edwards auto 500 FL 500, England). Finally, lift‐off was performed in acetone with the subsequent rinsing samples in isopropyl alcohol.

*PEDOT/MnO_2_ and PEDOT/Fe_3_O_4_ Preparation*: In order to easily achieve electrodeposition process on electrodes, samples were first fixed on special designed PCBs via VGE‐7031 adhesive with subsequent Au‐wire bonding for connecting the pads of device to PCBs outside. The above device was immersed in an aqueous electrolyte containing 10 × 10^−3^ m 3,4‐ethylenedioxythiophene (EDOT), 10 × 10^−3^ m sodium dodecyl sulfate, and 1 m H_2_SO_4_, followed by electrodeposition at a potential of 0.9 V with different time using platinum wire (a diameter of ≈1 mm, Autolab) and Ag/AgCl (saturated KCl) as counter and reference electrodes, respectively. Then, the PEDOT on interdigital electrodes was used as the scaffold for the growth of MnO_2_ and Fe_3_O_4_ for the cathode and anode. Initially, the electrochemical deposition was performed in 0.02 m FeCl_2_ solution at a constant voltage of −1.5 V for 5 min in the same three‐electrode cell. Similarly, the MnO_2_ nanosheets were also deposited via the electrodeposition method in an electrolyte containing 0.01 m Na_2_SO_4_ and Mn(CH_3_COO)_2_. Then, the electrodeposition of MnO_2_ was carried out using CV scan between 0.3 and 0.6 V (vs Ag/AgCl) at a scan rate of 50 mV s^−1^ for 12 cycles followed by a constant voltage of 0.6 V for 90 s, and this deposition process was repeated for three times. Finally, the samples were washed with distilled water.

*Rolled‐Up Self‐Assembly into Tubular Device*: The rolling process of planar devices was controlled by selectively etching the sacrificial layer in the solution of 0.5 m potassium diethylene triamine penta acetic acid (Alfa Aesar, UK) with pH 10 adjusted by potassium hydroxide. After rolling process completed, the 3D devices were continuously immersed in etching solution for 12 h to make the 3D architecture stable. Then, the devices were taken out of the solution and then sealed by 5 m LiCl/PVA gel electrolyte, which was prepared by mixing 2 g PVA powder and 4.24 g LiCl in 20 mL deionized water. Finally, the gel electrolyte outside tube was dried in air for two days.

*Materials Characterization*: The crystallographic information and chemical composition of as‐prepared products were established by powder XRD (Co Kα radiation (λ = 1.5418 Å), X'Pert PRO MPD, Philips). Raman analysis was performed by using a Raman spectrometer (LabRAM HR Evolution, HORIBA Scientific) at room temperature. The investigations of the samples' morphology were performed by SEM/FIB cross beam (Zeiss NVision 40). SEM imaging was done with electrons of 5 and 2 keV, respectively. FIB cuts were made using 30 keV Ga+ ions.

*Electrochemical Measurements*: The electrochemical properties including CV, GCD, and electrochemical impedance spectroscopy of the rolled‐up MSCs were carried out using an electrochemical workstation (µautolabIII/FRA2) in a 5 m LiCl/PVA gel electrolyte.

The areal specific capacitance (*C*) of the samples was calculated from the charge–discharge curves based on the following equation

(3) $$C=\frac{I\times \Delta t}{A\times \Delta V}$$

where *A*, *I*, ∆*t*, and ∆*V* are the footprint areal (cm^2^) of the tubular device, discharge current (mA), the discharging time (s), and the discharging potential range (V), respectively.

Energy density (*E*, Wh cm^−2^) and power density (*P*, W cm^−2^) of the devices were calculated using the following equations

(4) $$E=\frac{C\times \Delta V^{2}}{2}$$

(5) $$P=\frac{E}{\Delta t}$$

## Conflict of Interest

The authors declare no conflict of interest.

## Supporting information

Supplementary
