# Self‐Assembled Flexible and Integratable 3D Microtubular Asymmetric Supercapacitors

**DOI:** 10.1002/advs.202103927

**Published:** 2021-10-21

**Authors:** Fei Li, Jinhui Wang, Lixiang Liu, Jiang Qu, Yang Li, Vineeth Kumar Bandari, Daniil Karnaushenko, Christian Becker, Maryam Faghih, Tong Kang, Stefan Baunack, Minshen Zhu, Feng Zhu, Oliver G. Schmidt

*Adv. Sci*. **2019**, *6*, 1901051

DOI: 10.1002/advs.201901051

In the originally published article, two typing errors occurred for the unit of the energy density in the abstract and on page 7 last paragraph. The correct abstract and last paragraph of page 7 can be found below. The corrections do not affect any other interpretations and/or claims of the original article.

Abstract

The rapid development of microelectronics has equally rapidly increased the demand for miniaturized energy storage devices. On‐chip microsupercapacitors (MSCs), as promising power candidates, possess great potential to complement or replace electrolytic capacitors and microbatteries in various applications. However, the areal capacities and energy densities of the planar MSCs are commonly limited by the low voltage window, the thin layer of the electrode materials and complex fabrication processes. Here, a new‐type threedimensional (3D) tubular asymmetric MSC with small footprint area, high potential window, ultrahigh areal energy density, and long‐term cycling stability is fabricated with shapeable materials and photolithographic technologies, which are compatible with modern microelectronic fabrication procedures widely used in industry. Benefiting from the novel architecture, the 3D asymmetric MSC displays an ultrahigh areal capacitance of 88.6 mF cm^−2^ and areal energy density of 28.69 µW h cm^−2^, superior to most reported interdigitated MSCs. Furthermore, the 3D tubular MSCs demonstrate remarkable cycling stability and the capacitance retention is up to 91.8% over 12 000 cycles. It is believed that the efficient fabrication methodology can be used to construct various integratable microscale tubular energy storage devices with small footprint area and high performance for miniaturized electronics.

Page 7, last paragraph:

We have demonstrated novel on‐chip 3D tubular asymmetric MSCs based on PEDOT–MnO_2_ anode and PEDOT–Fe_3_O_4_ cathode materials employing Swiss‐roll architecture and shapeable materials technologies. The rolled‐up self‐assembly process can effectively reduce the footprint area of the original planar devices, greatly improving the areal specific capacitance (88.6 mF cm^−2^ at 0.66 mA cm^−2^) and areal energy density (28.69 µW h cm^−2^ at 0.25 mW cm^−2^). Moreover, the cycling stability of 3D tubular MSCs showed remarkable capacitance retention up to 91.8% over 12 000 cycles. In addition, the tubular MSCs show excellent mechanical performance without sacrificing their electrochemical storage properties. Our work demonstrates a new powerful strategy to create highly integrated MSCs with high performance and small footprint area. The 3D tubular MSC provides an efficient counterpart of conventional macroscopic cylinder‐shape energy storage devices at the microscale, and shows promising potential for microelectronic applications.

The authors apologize for any inconvenience this may have caused.

