# Supplementary material for: Self‐Assembled Flexible and Integratable 3D Microtubular Asymmetric Supercapacitors
Source: Adv Sci (Weinh). 2019 Aug 26;6(20):1901051. doi: 10.1002/advs.201901051 (PMC6794616; doi:10.1002/advs.201901051)
Supplement: Supplementary file 1 — Supplementary [file ADVS-6-1901051-s001.pdf]

Copyright WILEY-VCH Verlag GmbH & Co. KGaA, 69469 Weinheim, Germany, 2019.

ADVANCED SCIENCE
Open Access

Supporting Information

for *Adv. Sci.,* DOI: 10.1002/advs.201901051

Self-Assembled Flexible and Integratable 3D Microtubular Asymmetric Supercapacitors

Fei Li, Jinhui Wang, Lixiang Liu, Jiang Qu, Yang Li, Vineeth Kumar Bandari, Daniil Karnaushenko, Christian Becker, Maryam Faghih, Tong Kang, Stefan Baunack, Minshen Zhu, Feng Zhu, and Oliver G. Schmidt*

Supplementary Information

Self-Assembled Flexible and Integratable 3D Microtubular Asymmetric Supercapacitors

Fei Li, Jinhui Wang, Lixiang Liu, Jiang Qu, Yang Li, Vineeth Kumar Bandari, Daniil Karnaushenko, Christian Becker, Maryam Faghih, Tong Kang, Stefan Baunack, Minshen Zhu, Feng Zhu, Oliver G. Schmidt*

F. Li, J. Wang, L. Liu, J. Qu, Y. Li, V. K. Bandari, Dr. F. Zhu, Prof. O. G. Schmidt

Material Systems for Nanoelectronics

Chemnitz University of Technology

09107 Chemnitz, Germany

E-mail: f.zhu@ifw-dresden.de

F. Li, J. Wang, L. Liu, J. Qu, Y. Li, V. K. Bandari, Dr. F. Zhu, Prof. O. G. Schmidt

Center for Materials, Architectures and Integration of Nanomembranes (MAIN),

Chemnitz University of Technology

09126 Chemnitz, Germany

F. Li, J. Wang, L. Liu, J. Qu, Y. Li, V. K. Bandari, Dr. D. Karnaushenko, C. Becker, M. Faghih, T. Kang, S. Baunack, Dr.M. Zhu, Dr. F. Zhu, Prof. O. G. Schmidt

Institute for Integrative Nanosciences

Leibniz IFW Dresden

01069 Dresden, Germany

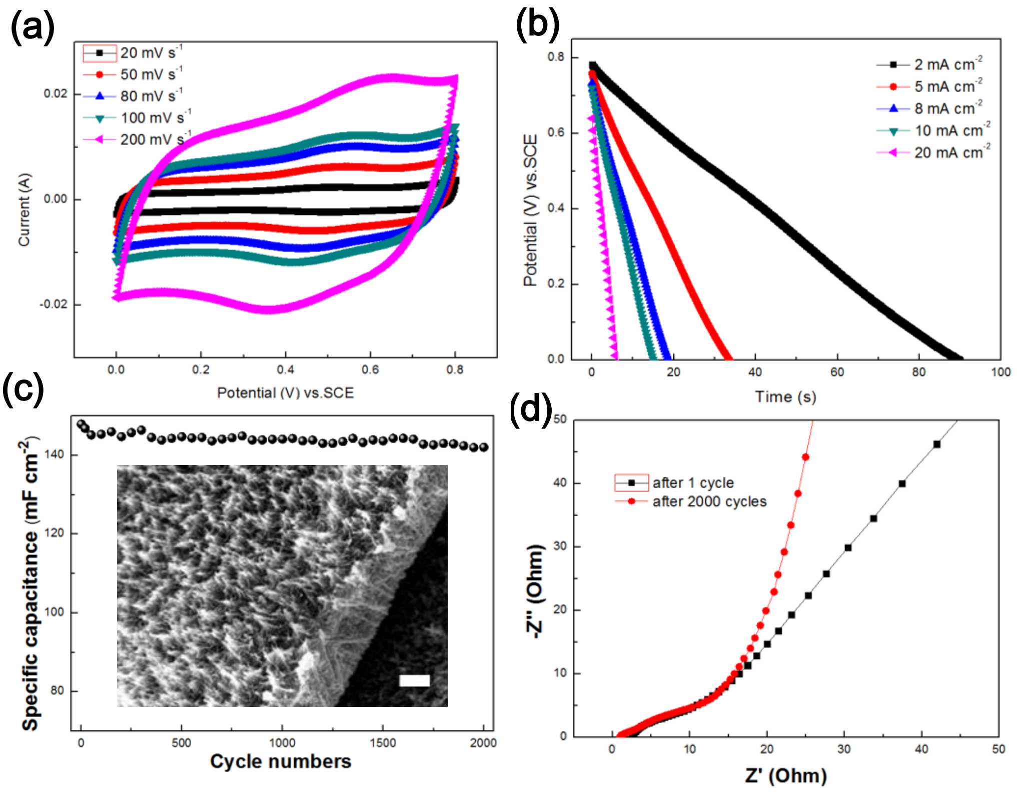

Figure S1. (a) CV curves with different scan rates. (b) GCD curves of MSCs at various current densities. (c) Cycling performance of 2000 cycles. Inset shows the SEM image of PEDOT-MnO_2 after cycling (Scale bar: 500 nm). (d) Electrochemical impedance spectra (EIS) after different cycles.

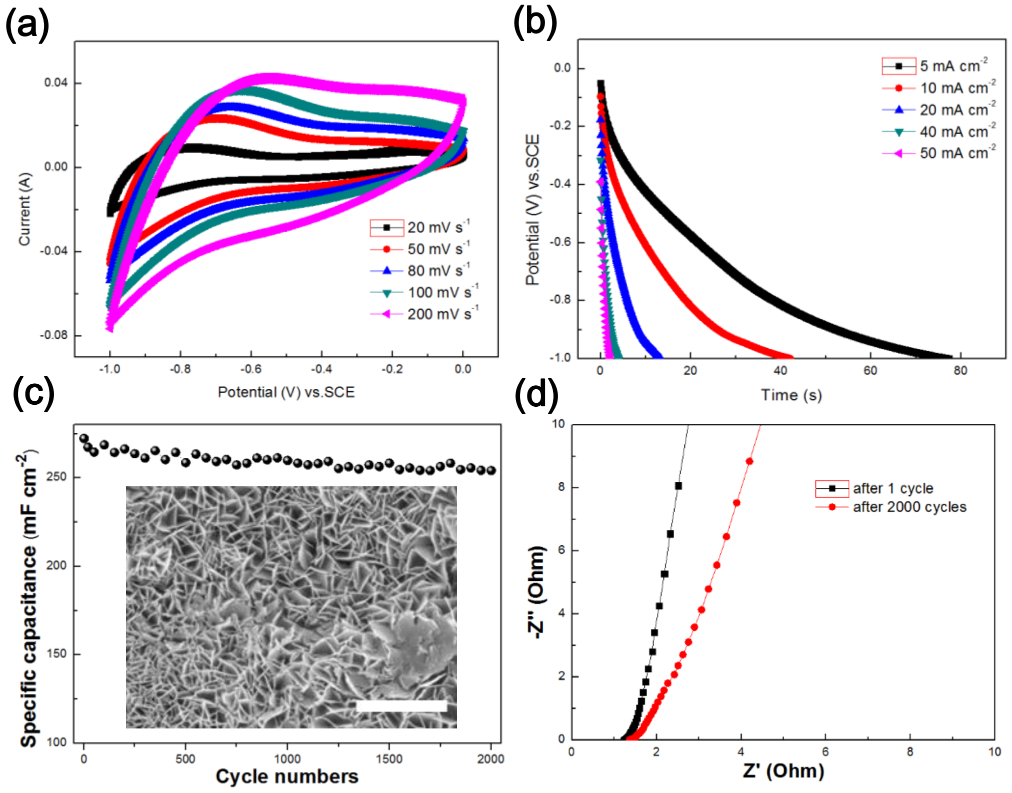

Figure S2. (a) CV curves with different scan rates. (b) GCD curves of MSCs at various current densities. (c) Cycling performance of 2000 cycles. Inset shows the SEM image of PEDOT- Fe_3O_4 after cycling (Scale bar: 2 μm). (d) Electrochemical impedance spectra (EIS) after different cycles.

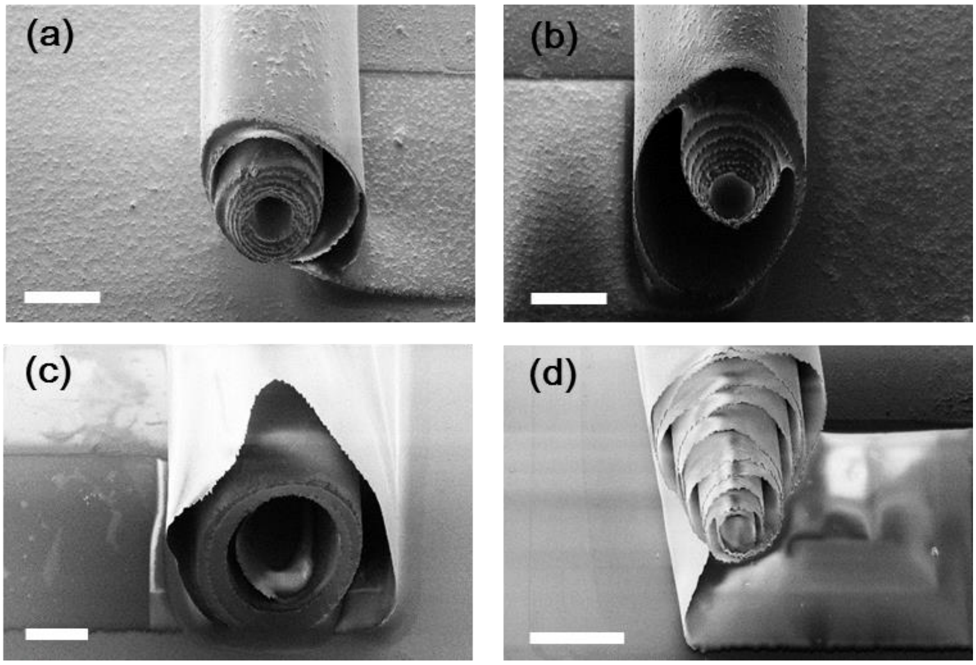

Figure S3. SEM images of the tube opening of different tubes (Scale bar (a-d): 100 μm).

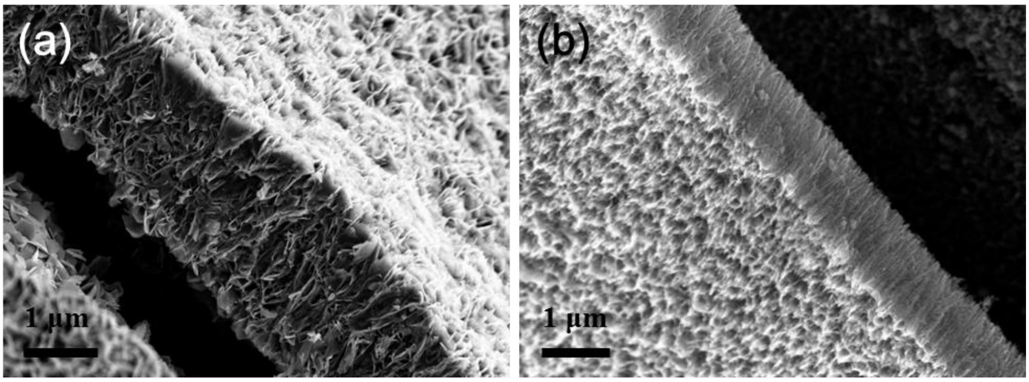

Figure S4. SEM images of the cross section of (a) PEDOT/Fe_3O_4 film and (b) PEDOT/MnO_2 film.

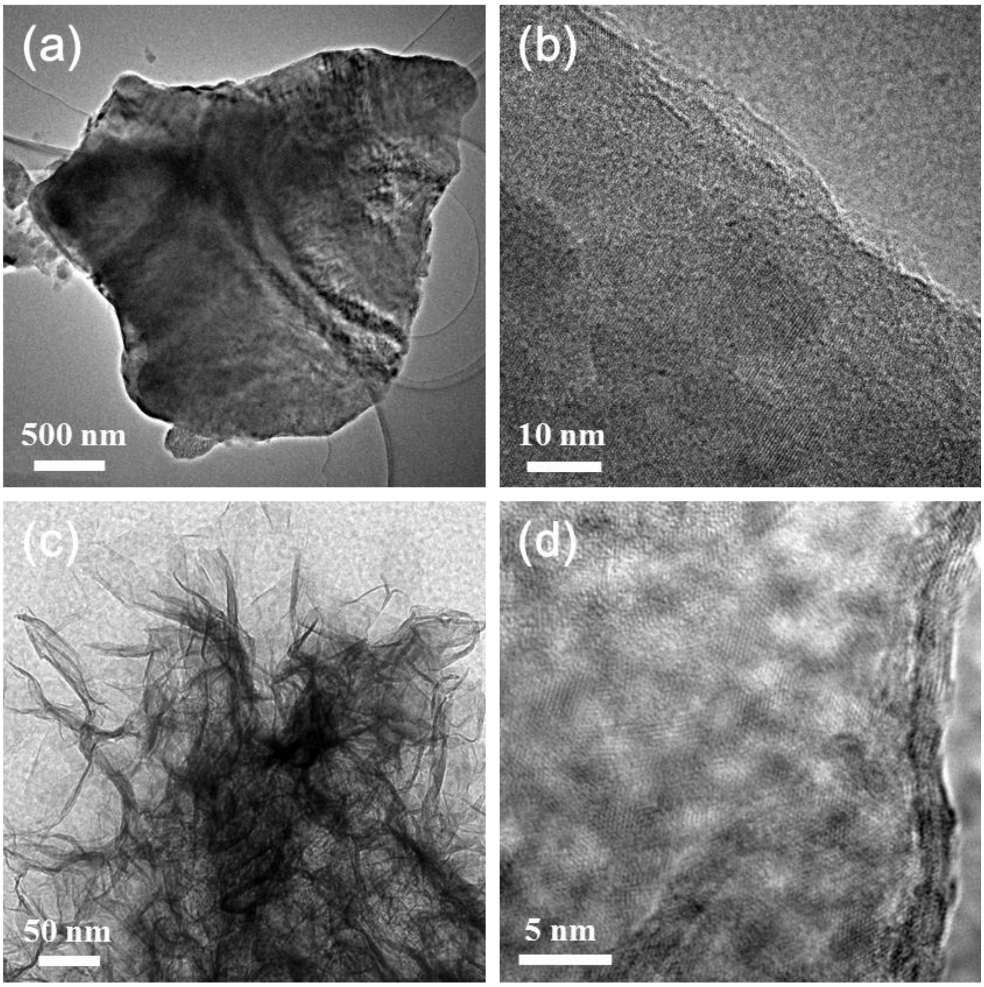

Figure S5. (a-b) TEM images of the Fe_3O_4 nanostructures; (c-d) TEM images of MnO_2 nanostructures.

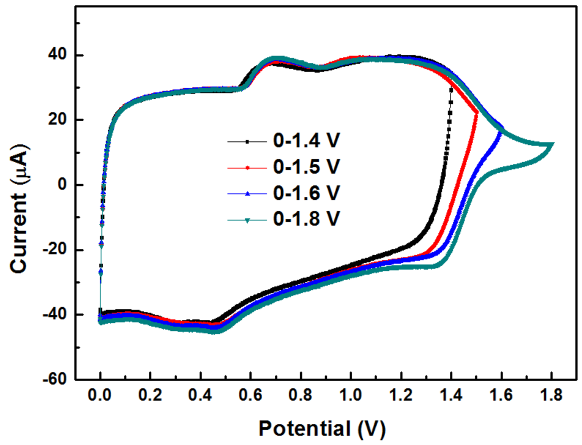

Figure S6. CV curves of MSCs tested in different voltage windows.

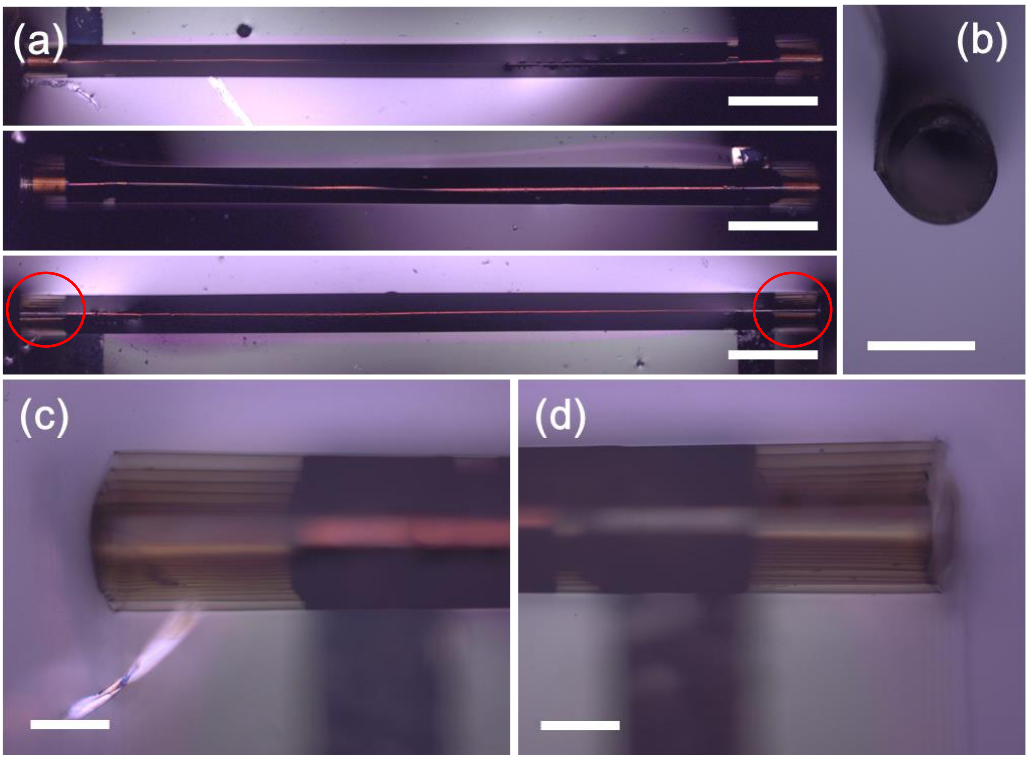

Figure S7. Optical microscope images of the tubes after a long term cycling: (a) three different tubes; (b) cross section of one end; (c-d) zoom-in pictures of the top view of two ends. (Scale bar (a): 500 μm; (b): 200 μm; (c): 100 μm; (d): 100 μm).

Table S1. Electrochemical performance of typical in-plane MSCs.

MSC	Electrolyte	Voltage	Footprint area	Areal capacitance	Cycling stability	Energy density	Ref.
$Cu(OH)_2$@FeOOH	[EMIM][BF4]/SiO_2	0-1.5 V	64 mm^2	58.0 mF cm^{-2}	82% (10000)	18.07 μW h cm^{-2}	11
LSG–MnO_2 //LSG	1 M Na_2SO_4	0-2.0V	24 mm^2	400 mF cm^{-2}	96% (10000)	22-42 Wh L^{-1}	23 a
graphite oxide LSG//LSG	PVA/H_2SO_4	0-1 V	200 mm^2	2.32 mF cm^{-2}	96% (10000)	-	2
B-3D-PCP	1.0 M H_2SO_4	0-1 V	8 mm^2	7.15 mF cm^{-2}	98% (30000)	7.1 mWh cm^{-3}	23b
LSG//LSG	1 M H_2SO_4/PVA	0-1 V	300 mm^2	25.1 mF cm^{-2}	98.5% (12000)	2.6 μWh cm^{-2}	24
Ti3C2Tx //Ti3CNTx	PVA/H_2SO_4	0-0.6 V	300 mm^2	61 mF cm^{-2}	94.1% (10000)	0.63 μWh cm^{-2}	25
PEDOT // PEDOT	1 M H_2SO_4	0-0.8 V	2400 mm^2	9 mF cm^{-2}	80% (10000)	7.7 mW h cm^{-3}	23c
VOx/rGO//G–VNQDs/rGO	5 M LiCl–PVA gel	0-1.6 V	16.32 mm^2	207.9 mF cm^{-2}	65% (8000)	73.9 μWh cm^{-2}	14
carbon-nanotubes (CNTs)	PVA-H_3PO_4	0-1 V	3 mm^2	2.44 mF cm^{-2}	93% (2000)	3.72 W cm^{-3}	23d
EG//EG	PVA/H_2SO_4	0-1 V	600 mm^2	5.4 mF cm^{-2}	90% (5000)	-	23e
polypyrrole/C//polypyrrole/C	0.1 M KCl	0-0.8 V	70 mm^2	78.35 mF cm^{-2}	56% (1000)	-	9
MnO_2	1MNa_2SO_4	0-0.8 V	1.87 mm^2	56.3 mF cm^{-2}	72.5% (1000)	22.95 μW h cm^{-2}	10
rGO–CNT // rGO–CNT	3 M KCl	0-1 V	7.5 mm^2	6.1 mF cm^{-2}	95 % (1000)	0.68 mWh cm^{-3}	7
EG/V_2O_5-MSC	PVA/LiCl gel	0-1 V	250 mm^2	3.92 mF cm^{-2}	93 % (6000)	20 mWh cm^{-3}	23f
onion-like carbon	1 M Et_4NBF_4/APC	0-3 V	25 mm^2	0.9 mF cm^{-2}	-	1*10^{-2} Wh cm^{-3}	8
PEDOT-MnO_2// PEDOT-Fe_3O_4	PVA/LiCl gel	0-1.5 V	0.76 mm^2	88.6 mF cm^{-2}	91.8% (12000)	28.69 μW h cm^{-2}	This work

Table S2. Mechanical performance, specific capacitance and cycling stability of different types of MSCs.

**MSC**	**Structure**	**Bending**	**Twisting /Stretching**	**Capacitance**	**Cycling stability**	**Ref.**
Ti_3C_2Tx MXene	Planar (interdigital)	NA	NA	0.5 mF cm^{-2}	NA	28a
δ-MnO_2	Planar (interdigital)	0 º-120 º	NA	0.26 mF cm^{-2}	78% (3600)	28b
MnOx–MHCF//AC	Planar	0 º-180 º	NA	175 mF cm^{-2}	94.5% (10000)	28c
$K_2Co_3(P_2O_7)_2{\cdot}{}_2H_2O$//graphene	Planar (interdigital)	0 º-180 º	NA	6.0 F·cm^{-3}	94.4% (5000)	28d
LIG/PDMS	Fiber	0 º-160 º	50% stretching	650 μF cm^{-2}	96% (10000)	28e
CNT@MnO_2 /CNT@PPy	Fiber	NA	20% stretching	60.43 mF cm^{-2}	80% (5000)	28f
CNT fibers and sheets	Fiber	0 º-180 º	10% stretching	8.66 mF cm^{-2}	100% (11000)	28g
RGO-CNT@CMC	Fiber	0 º-180 º	NA	269 mF cm^{-2}	100% (2000)	28h
Ti_3C_2Tx MXene	3D	NA	NA	34.6 mF cm^{-2}	91% (15000)	28i
graphene	3D	NA	NA	74.31 mF cm^{-2}	100% (10000)	28j
carbon-nanotubes	3D	NA	NA	2.44 mF cm^{-2}	93% (1500)	23a
VOx/rGO//G–VNQDs/rGO	3D	NA	NA	207.9 mF cm^{-2}	65% (8000)	14
PEDOT-MnO_2// PEDOT-Fe_3O_4	3D	0 º-90 º	0 º-60 º twisting	88.6 mF cm^{-2}	91.8% (12000)	This work
